# Novel sulphonamide-bearing methoxyquinazolinone derivatives as anticancer and apoptosis inducers: synthesis, biological evaluation and *in silico* studies

**DOI:** 10.1080/14756366.2021.1983807

**Published:** 2021-12-11

**Authors:** Ali S. Alqahtani, Mostafa M. Ghorab, Fahd A. Nasr, Mohammad Z. Ahmed, Abdullah A. Al-Mishari, Sabry M. Attia

**Affiliations:** a Department of Pharmacognosy, College of Pharmacy, King Saud University, Riyadh, Saudi Arabia; b Medicinal, Aromatic and Poisonous Plants Research Center, College of Pharmacy, King Saud University, Riyadh, Saudi Arabia; c Department of Drug Radiation Research, National Center for Radiation Research and Technology (NCRRT), Egyptian Atomic Energy Authority (EAEA), Cairo, Egypt; d Department of Pharmacology and Toxicology, College of Pharmacy, King Saud University, Riyadh, Saudi Arabia

**Keywords:** Quinazolinone, benzenesulfonamide, cytotoxicity, caspase, apoptosis

## Abstract

We synthesised a new series of sulphonamide-bearing quinazolinone derivatives **5-18** and evaluated their *in vitro* cytotoxicity in various cancer cell lines (A549, HepG-2, LoVo and MCF-7) and in normal human cells (HUVEC). Compounds **6** and **10** exhibited the higher activity against all the cancer cell lines compared with 5-flourourcil as positive control. The ability of the most promising compounds **6** and **10** to induce cell cycle arrest and apoptosis in breast cancer (MCF-7) cells was evaluated by flow cytometry. Reverse transcriptase-polymerase chain reaction and western blotting were used to evaluate the expression of apoptosis-related markers. We found that the 2-tolylthioacetamide derivative **6** and the 3-ethyl phenyl thioacetamide derivative **10** exhibited cytotoxic activity comparable to that of 5-fluorouracil as reference drug in MCF-7 and LoVo colon cancer cells. Cell cycle analysis showed a concentration-dependent accumulation of cells in the sub-G1 phase upon treatment with both compounds. The Annexin V-fluorescein isothiocyanate/propidium iodide assay showed that the compounds **6** and **10** increased the early and late apoptosis cell death modes in a dose-dependent manner. These compounds downregulated the expression of B-cell lymphoma-2 (Bcl-2), while increasing that of p53, Bcl-2-like protein 4, and caspase-7, at the mRNA and protein levels. Molecular docking of compounds **6** and **10** with Bcl-2 predicted them to show moderate − high binding affinity (**6**: −7.5 kcal/mol, **10**: −7.9 kcal/mol) and interactions with key central substrate cavity residues. Overall, compounds **6** and **10** were found to be promising anticancer and apoptosis-inducing agents.

## 1. Introduction

**Figure 1.**
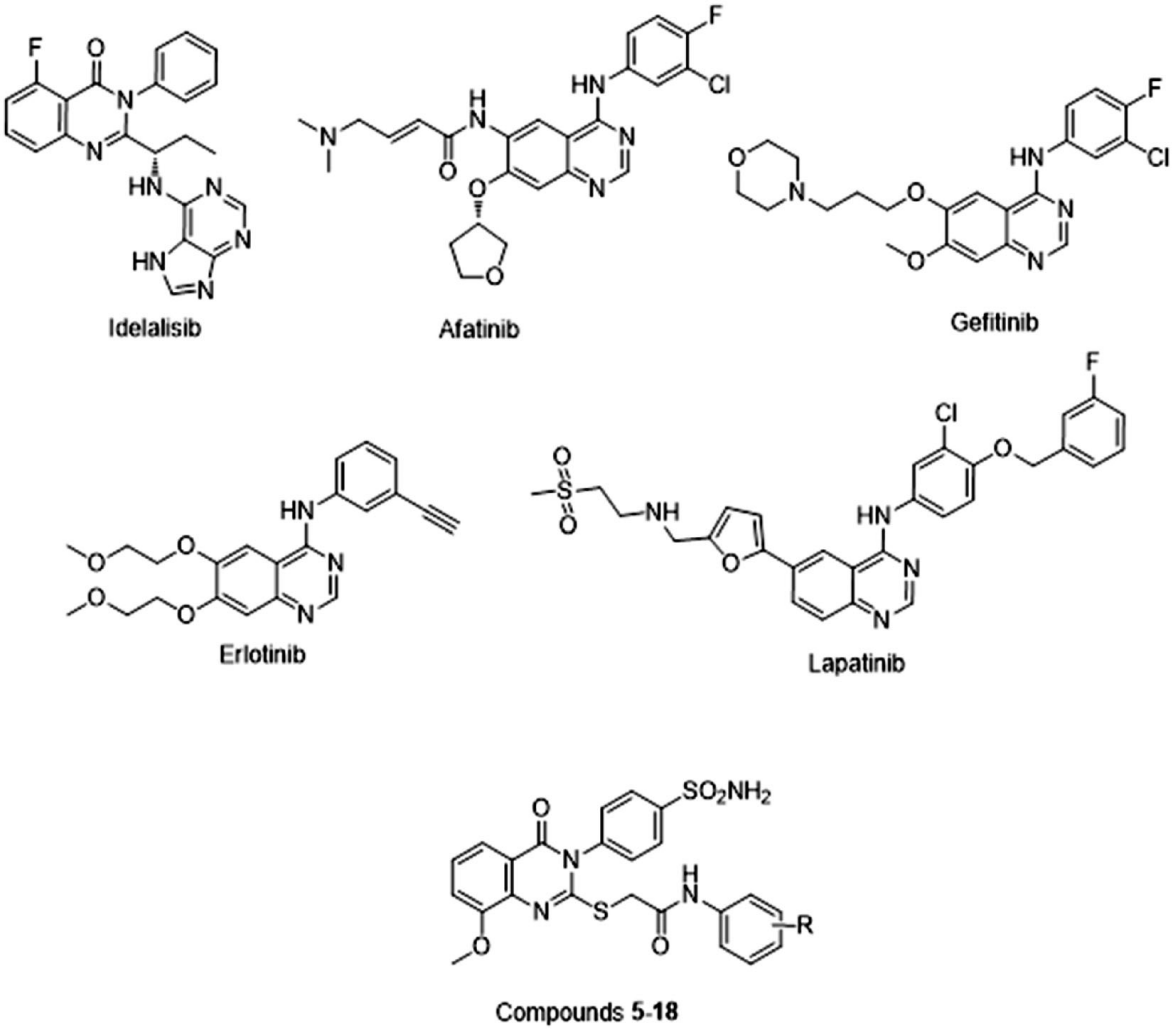
Quinazoline-containing drugs.

Cancer is a life-threatening disease that is considered a major medical challenge worldwide^1^. Treatments for cancer include surgery, chemotherapy, hormonal therapy, and biological therapy^2–4^. The choice of treatment is influenced by the site and progression of the disease. Chemotherapy is primarily used for the treatment of metastasis and hypoxic tumours. However, its use is limited by the toxicity of the drugs towards healthy cells^5^^,^^6^. This toxicity is a result of the low selectivity of existing chemotherapeutic drugs towards cancer cells^7^. In addition, the long-term use of chemotherapeutic agents often gives rise to drug resistance^8^. The continuous search for new anticancer agents that offer both selectivity towards malignant cells and low potential for resistance is required. Interest in quinazolinone derivatives grew after showing numerous activity in medical chemotherapy such as apoptosis induction and antiangiogenic properties. Idelalisib, afatinib, gefitinib, erlotinib, and lapatinib (Figure 1) are among these compounds that have been reported to exert apoptosis induction and cell cycle arrest in different cancer cells^9–14^. The main factors contributing to the interest in these compounds are their good safety profile and potential for oral administration^15^^,^^16^. Hybridisation has proven to be beneficial in the preparation of new anticancer agents and in overcoming the drawbacks of conventionally used drugs^17^^,^^18^. Therefore, sulphonamides were hybridised with quinazolin-4(*3H*)-ones to obtain potentially better drug candidates. Sulphonamides mimic the properties of p-aminobenzoic acid and block SH- and NH_2_-containing enzymes and proteins to exhibit antimicrobial activity^19^^,^^20^.

We designed a series of novel compounds using the quinazolinone-sulphonamide hybrid scaffold and explored their *in vitro* cytotoxic effects against a range of cancer cell lines. The hybrid group was fixed and structural modifications focussed on the replacement of the thiol group at the C-2 position of quinazolines with thioacetamide derivatives bearing substituted phenyl rings. The activities of the target compounds were evaluated against MCF-7, HepG-2, LoVo, and A549 cancer cell lines; the most potent compounds were then evaluated for their pro-apoptotic activity, in order to analyse the underlying anticancer mechanisms. To explore the possible pharmacological properties involved in the anticancer activity, the cell cycle and apoptosis were evaluated by flow cytometry. The target compounds were established to initiate apoptosis, which synergistically enhanced the antitumor effects^21^^,^^22^. Therefore, the apoptotic effects of the most potent compounds were explored by evaluating caspase-7 activity and by monitoring B-cell lymphoma-2 (Bcl-2) and Bcl-2-like protein 4 (Bax) levels using *in vitro* and *in silico* techniques.

## 2. Results and discussion

### 2.1. Chemistry

**Scheme 1.**
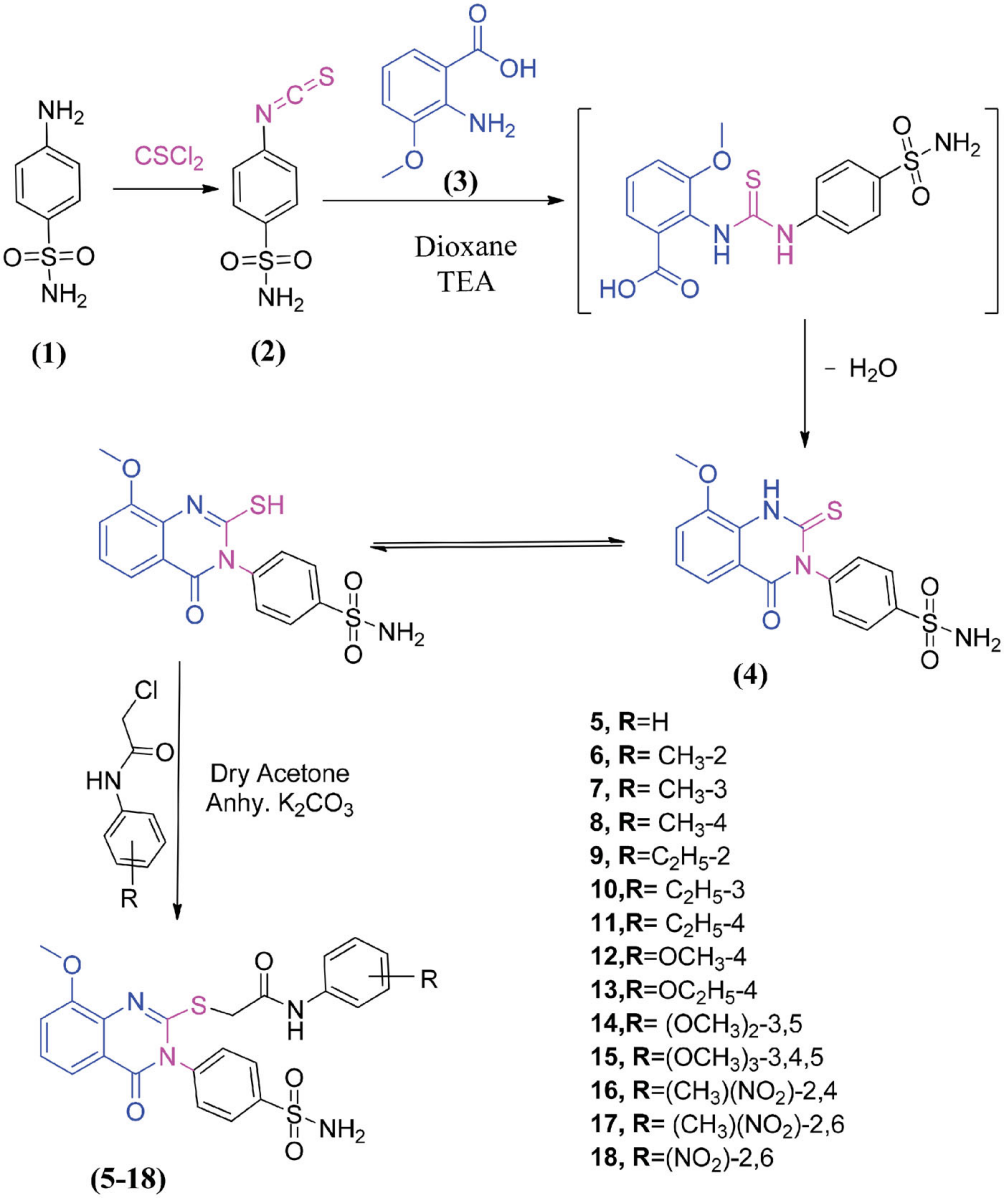
Synthesis of the sulphonamide bearing methoxyquinazolinone **4-18**.

In this work, it seemed of interest to search for new heterocyclic compounds with anticancer activity. A novel series of 3,4-dihydroquinazolinone conjugated to a biologically active benzenesulphonamide moiety was synthesised by the introduction of benzenesulphonamide at the 3-position with the incorporation of different types of acetamide terminal at 2-position aimed at exploring the potential anticancer activity. Scheme 1 shows the synthesis of quinazolinone-benzenesulphonamide derivatives **5–18**. The starting material, *4*-(2-mercapto-4-oxoquinazolin-3(*4H*)-yl) benzenesulphonamide (**4**) was prepared in quantitative yield by cyclocondensation of 4-isothiocyanatobenzenesulfonamide (**2**)^23^ and 2-amino-3-methoxybenzoic acid (**3**) in refluxing 1,4-dioxan containing a catalytic amount of triethylamine. The structure of compound **4** was characterised from correct analytical data as well as its infra-red (IR) spectrum which showed a characteristic bands at 3321, 3262, 3181 cm^−1^ (NH_2_), 3055 cm^−1^ (CH aromatic), 1691 cm^−1^ (CO), 1620 cm^−1^ (CN), 1381, 1156 cm^−1^ (SO_2_). ^1^H-NMR spectrum exhibited signals at 3.9 ppm attributed to OCH_3_ and 12.3 ppm assigned to SH group. ^13 ^C- NMR spectrum revealed signals at 55.7 ppm due to OCH_3_, 160.5 ppm for C=N and 160.9 ppm attributed to C=O. The coupling of **4** and 2-chloro-*N*-substituted acetamides in dry acetone, in the presence of anhydrous K_2_CO_3_ at room temperature yielded the corresponding 2-((8-methoxy-4-oxo-3-(4-sulfamoylphenyl)-3,4-dihydroquinazolin-2-yl)thio)-*N*-substituted phenyl acetamides **5–18**. The synthesised compounds **5–18** were characterised on the basis of their spectral data. The IR spectra of compounds **5–18** displayed additional NH, NH_2_, CH aromatic, CH aliphatic, 2CO, CN and SO_2_ characteristic bands in the assigned regions. Proton nuclear magnetic resonance (^1^H-NMR) spectra of compounds **5–18** revealed two singlet signal peaks (3.9 − 4.1 ppm, representing the CH_2_; 7.9 − 10.3 ppm, representing the NH) and the loss of the SH singlet of **3**. The ^13 ^C-NMR spectra of compounds **5–18** showed two signals peculiar to the CH_2_ and CO carbons. The ^1^H-NMR spectra of compounds **6–8** showed singlet peaks at 2.4, 2.2 and 2.3 ppm, which were attributed to the CH_3_ groups at the *ortho*, *meta* and *para-* positions of the phenyl group, respectively. The ^1^H-NMR and ^13 ^C- spectra of compounds **9–11** showed triplets (1.0, 1.1, 1.1 ppm, respectively) and quartettes at 2.5 ppm, attributed to the CH_3_ and CH_2_, respectively of the ethyl groups, at the *ortho*, *meta* and *para* positions of the phenyl ring. The ^13 ^C-NMR spectra of compounds **9–11** showed signals corresponding to the CH_3_ (14.5, 15.9 and 16.1 ppm, respectively) and CH_2_ (24.0, 28.7, and 28.0, respectively) of the ethyl groups. The IR spectra of compounds **16–18** showed bands corresponding to the NO_2_ groups in the specified region. The ^1^H-NMR spectra of **16** and **17** showed singlets at 2.3 and 2.2 ppm, respectively, due to the CH_3_ group, while the ^13 ^C-NMR showed signals at 19.3 and 18.0 ppm, respectively.

### 2.2. Biological evaluation

#### 2.2.1. Cytotoxicity screening

The anticancer activity of compounds **5–18** was evaluated against a panel of cell lines, including non-small cell lung carcinoma (A549), liver (HepG2), colon (LoVo), and breast (MCF-7) cancer cell lines, as well as normal human umbilical vein endothelial cells (HUVEC), by 3-(4,5-dimethylthiazol-2-yl)-2,5-diphenyl tetrazolium bromide (MTT) assay. 5-Fluorouracil was used as reference drug. As shown in Table 1, the screening results indicated that eight compounds, namely 3, 4-dihydroquinazolin-sulphonamide carrying 2-tolylthioacetamide group at 2-position **6,** 3- tolyl **7,** 3-ethylphenyl **10,** 4-ethylphenyl **11**, 4-ethoxyphenyl **13,** 3,5-dimethoxyphenyl **14,** 3,4,5-trimethoxyphenyl **15** and 2-methyl-4-nitrophenyl **16** exhibited powerful cytotoxic activity against all cancer cell lines compared to 5-flourouracil as positive control. In general, all the tested compounds tended to exhibit better efficacy against MCF-7 cells than against the other cell lines. Among the compounds screened, the 2-tolyl derivative **6** and the 3-ethylphenyl derivative **10** exhibited the most promising activity. The 2-tolyl derivative **6** exhibited good antitumor activity, with half maximal inhibitory concentrations (IC_50_ values 20.17, 22.64, 45. 57, and 51.50 µM, against MCF-7, LoVo, HepG2, and A549 cell lines, respectively. Interestingly, 2-tolyl derivative **6** was found to be much less cytotoxic towards normal HUVECs (IC_50_=88.27 µM). The 3-ethylphenyl derivative **10** also exhibited good cytotoxic potency against MCF-7 cells (IC_50_ = 20.91 µM), LoVo (IC_50_ = 22. 30 µM), HepG2 (IC_50_ = 42.29 µM), and A549 (IC_50_ = 48.00 µM). The presence of the methyl group at 2-position in phenyl ring in compound **6** with IC_50_ value = 20.17 µM and ethyl group at 3-position in phenyl ring in compound **10** with IC_50_ value = 20.91 µM lead to an increase in the anticancer activity against human breast cancer cell line MCF-7 compared to the 5-flourouracil as reference drug with IC_50_ value 95.63 µM. It is clear from the present data that the comparison of the IC_50_ for the synthesised compounds against human breast cancer cell line MCF-7 follows the order **6 **>** 10 **>** 7** > **11 **>** 14 **>** 16 **>** 13 **>** 15 **>** **5-fluorouracil with IC_50_ values 20.17, 20.91, 27.69, 29.40, 34.60, 39.90, 46.72, 74.03, 95.63, respectively. On the other hand, compounds **5**, **8**, **9**, **12**, **17** and **18** showed no activity towards all the cell lines. Based on the MTT screening results, compounds **6** and **10** were found to be the most potent against MCF-7 cells, so they were pursued for further investigation.

**Table 1.** *In vitro* anticancer activities of compounds **5–18** against A549, HepG2, LoVo, MCF-7 and HUVEC cell lines.

Compound No.	Cell lines and IC_50_ (µM)				
	A549	HepG2	LoVo	MCF-7	HUVEC
**5**	NA	NA	NA	NA	NA
**6**	51.5 ± 1.36	45.57 ± 0.71	22.64 ± 0.86	20.17 ± 0.6	88.27 ± 0.32
**7**	58.55 ± 1.52	39.69 ± 2.27	30.1 ± 1.3	27.69 ± 1.85	83.15 ± 0.9
**8**	NA	NA	NA	NA	NA
**9**	NA	NA	NA	NA	NA
**10**	48 ± 2.78	42.29 ± 1.59	22.3 ± 2.26	20.91 ± 0.4	32 ± 1.47
**11**	50.5 ± 1.73	42.67 ± 0.84	47 ± 1. 6	29.4 ± 0.33	32.93 ± 1.4
**12**	NA	NA	NA	NA	NA
**13**	61.87 ± 1.35	38 ± 0.47	54.38 ± 2.07	46.72 ± 1.23	38.99 ± 1.4
**14**	68.34 ± 0.42	72.45 ± 1.06	38.26 ± 0.65	34.6 ± 1.15	62.55 ± 2.34
**15**	75 ± 1.74	75.25 ± 0.96	47.52 ± 4.08	74.03 ± 2.16	78.97 ± 1.09
**16**	67.4 ± 1.45	75.74 ± 1.85	40.98 ± 0.12	39.9 ± 0.57	80.78 ± 1.13
**17**	NA	NA	NA	NA	NA
**18**	NA	NA	NA	NA	NA
**5-Flurouracil**	98.86 ± 0.01	132.38 ± 0.6	90.71 ± 0.5	95.63 ± 0.3	166.97 ± 0.2

*(NA): No activity. Values represent the mean ± standard deviation (*n* = 3).

#### 2.2.2. Cell cycle analysis

**Figure 2.**
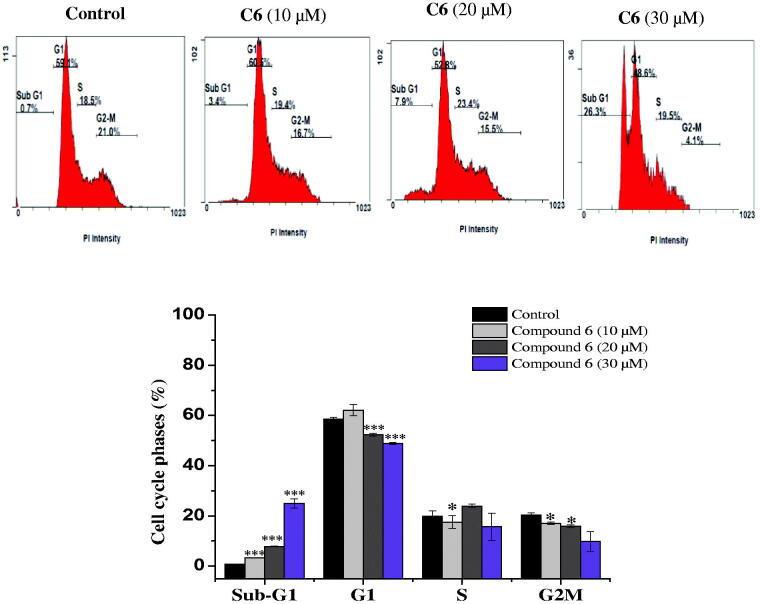
Cell cycle distribution of MCF-7 cells analysed by flow cytometry after treatment with compound 6 at (10, 20 and 30 μM) concentrations for 24 h. The histogram showing the cells percentage in control and treated cells. Columns, average ± SD. *p < 0.05, **p < 0.01 and ***p < 0.001 as compared to control.

**Figure 3.**
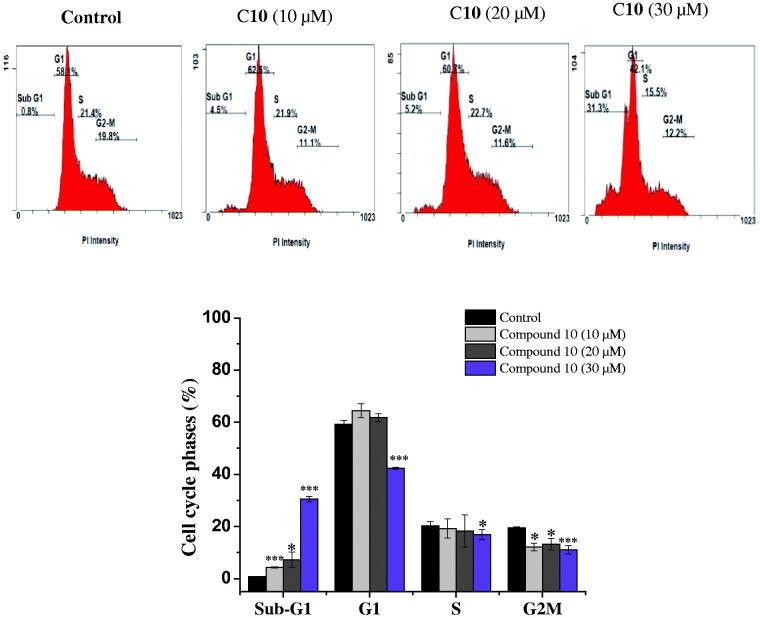
Cell cycle analysis of MCF-7 cells after treatment with compound **10**. MCF-7 cells were treated with different concentrations of compound **10** (10, 20, 30 μM) for 24 h, stained with propidium iodide and analysed for cell cycle using flow cytometer. Columns, average ± SD. *p < 0.05, **p < 0.01 and ***p < 0.001 as compared to control.

The capacity of anticancer drugs to influence cell cycle distribution can provide an insight into the mechanism of their cytotoxic activity^24^. In fact, several cell cycle inhibitors have emerged as prospective therapeutic medications for the treatment of several tumours^25^. Following the cytotoxicity screening, the effect of the most active compounds **6** and **10** on cell cycle progression in the MCF-7 cell line was evaluated. Compared with untreated cells, MCF-7 cells treated with compounds **6** and **10** had a significantly higher percentage of cells in the sub-G1. This increase in sub-G1 phase cells exhibited dose-dependence. Treatment with 10, 20, and 30 µM of the promising compound **6** increased the proportion of sub-G1 phase cells to 3.35 ± 0.07%, 7.8 ± 0.14%, and 22.85 ± 1.2%, respectively, versus the control (0.75 ± 0.07%) (Figure 2). Similarly, treatment of MCF-7 cells with the active compound **10** (10, 20, and 30 µM) also caused an accumulation of cells in the sub-G1 phase (4.3 ± 0.28%, 7.25 ± 2. 89% and 30.6 ± 0.07%, respectively), compared to the control (0.75 ± 0.07%) (Figure 3). This increase in the proportion of sub-G1 phase cells was accompanied by a significant decrease in the percentage of cells in the G1 and G2-M phases. It has been proposed that the increment in the sub-G1 cell fraction is indicative of apoptotic cell death^26^, suggesting that both the biologically active compounds **6** and **10** induced apoptosis in MCF-7 cells.

#### 2.2.3. Quantification of apoptosis

**Figure 4.**
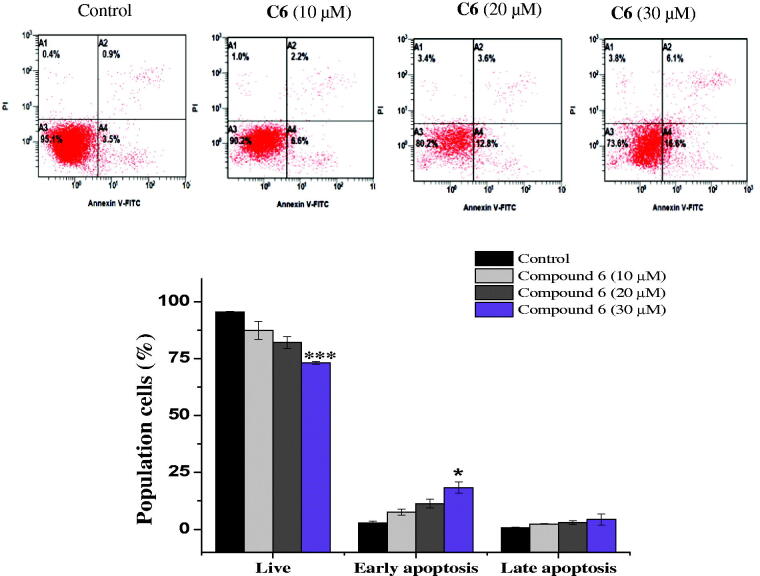
Apoptosis detection in MCF-7 cells using annexin V − FITC/PI double-staining assay. Cells were treated with 10, 20 and 30 μM of C6 for 24 h. Dot plots: Necrotic cells (A1), live cell (A2), late apoptotic cells (A3) and early apoptotic cells (A4). The quantitative analyses of apoptotic cells are shown in bar graphs. Data are expressed as average ± SD of three experiments.

**Figure 5.**
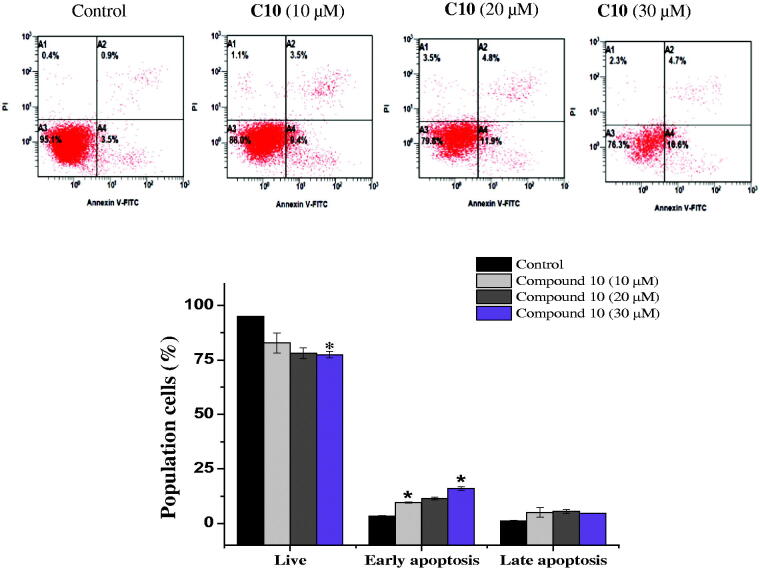
Effect of compound **10** on apoptosis induction of MCF-7 cells treated with various concentrations of **C10** (10, 20 and 30 μM) for 24 h. Apoptosis quantification was performed by Annexin V-FITC/PI dual staining assay. Data represents replicates of three experiments, and is expressed as the average ± SD. Data was analysed using the Student’s t test.

Apoptosis evasion is also a hallmark of the transformation of normal cells into tumour cells^27^. Common anticancer drugs aim to induce cell death through apoptosis; this is viewed as a requirement for blocking malignant cell growth^28^. To verify that apoptotic cell death was caused by the promising compounds **6** and **10**, an Annexin V-fluorescein isothiocyanate (FITC)/propidium iodide (PI) assay was used to quantify the cells undergoing apoptosis. In addition to the accumulation of cells in the sub-G1 phase, the Annexin V/PI assay has been widely utilised for the detection of apoptotic cells^29^. As shown in Figure 4, the treatment of MCF-7 cells with compound **6** (10, 20, and 30 µM) for 24 h cells increased the proportion of cells in early apoptosis (7.6 ± 1.4%, 11.4 ± 1.97%, and 18.35 ± 2.4%, respectively), compared to the control group (2.9 ± 0.84%). Similarly, the number of early apoptotic MCF-7 cells increased to 9.55 ± 0.2%, 11.4 ± 0. 7%, and 16 ± 0.84% on 24 h-treatment with 10, 20, and 30 µM compound **10**, respectively, compared to the control group (3.3 ± 0.28%). An increase in the number of late apoptotic cells was also observed (Figure 5). Overall, the flow cytometry data suggested that both compounds induced cell death through the induction of apoptosis, in a dose-dependent manner.

#### 2.2.4. Expression of p53, bax, caspase-7 and bcl-2

**Figure 6.**
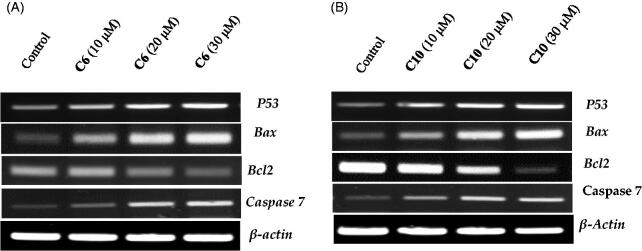
Evaluation of the effects of compound **6 (A)** and compound **10 (B)** on the expression of p53, Bax, caspase-3 and Bcl-2 using reverse transcriptase-polymerase chain reaction. MCF-7 cells were cultured and treated in the presence and absence of compound **6** (10, 20, and 30 μM) (A) and compound 10 (10, 20, and 30 μM) (B). β-actin was used as the internal control.

Next, we explored whether the induction of apoptosis caused by the active compounds **6** and **10** was associated with the activation of apoptosis-related genes. Members of the Bcl-2 family, especially the pro-apoptotic Bax and anti-apoptotic Bcl-2 genes, are known to play a crucial role in the regulation of the apoptotic pathway. The Bax and Bcl-2 genes participate in the downstream initialising of caspase proteins^30^. Therefore, the expression of key genes and the levels of proteins that control the apoptosis pathway were investigated to evaluate the pro-apoptotic effect of the compounds. The expression of p53, Bax, caspase-7, and Bcl-2 was evaluated using specific primers and antibodies. β-actin was used as an internal control. A remarkable change in the expression of apoptotic genes was reported after 24 h of treatment with increasing concentrations of the two compounds. The expression of p53, Bax, and caspase-7 mRNA increased as compared to the control, when the concentrations of compounds **6** and **10** were increased (Figure 6(A,B)). Meanwhile, the expression of Bcl-2 was downregulated with increasing doses of compounds **6** and **10**, as compared to that of the control.

**Figure 7.**
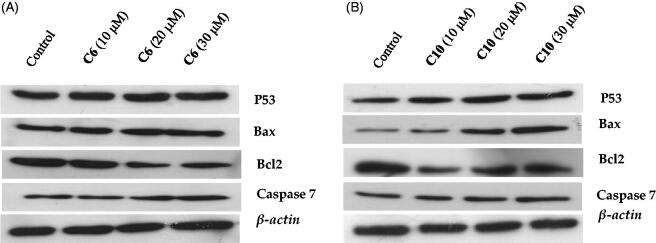
Evaluation of the effects of compound **6** (A) and compound **10** (B) on the protein levels of p53, Bax, caspase-7 and Bcl-2 by western blotting. MCF-7 cells were cultured and treated in the presence and absence of compound **6** (10, 20, and 30 μM) (A) and compound **10** (10, 20, and 30 μM) (B). β-actin was used as the internal control.

To assess the changes in expression of p53, Bax, Bcl-2, and caspase-7 proteins upon treatment with the compounds, measurements of the protein levels in MCF-7 cells treated with different dosed of compounds **6** and **10** were carried out. Western blot analysis revealed that treatment with compounds **6** and **10** markedly increased the levels of p53, Bax, and caspase-7 (Figure 7(A,B)). In contrast, the expression of Bcl-2 was down-regulated; this shift in the Bax/Bcl-2 ratio corresponds with the onset of cell apoptosis^31^. The activation of caspase-7 was also evident (Figure 7), indicating the initiation of cellular apoptosis by both compounds.

### 2.3. Structure–activity relationship

By comparing the experimental cytotoxicity of the novel synthesised compounds reported in this study to their structures, the following structure–activity relationships (SAR) were postulated.

- The presence of a basic skeleton quinazolin-sulphonamide is necessary for the broad spectrum of cytotoxic activity of the most compounds namlly **6**, **7**, **10**, **11**, **13**–**16** towards different cell lines (A 549, HepG2, LoVo and MCF-7).
- Introducing 2-tolyl at 2- position with the biologically active benzenesulphonamide moiety at 3- position and methoxy group at 8- position to the quinazoline **6** enhances the cytotoxic activity against all the cell lines.
- Also, the incorporating 3-ethylphenyl at 2- position to quinazoline-sulphonamide **10** lead to increase the cytotoxicity towards all cell lines.
- Transformation of 2-tolyl in compound **6** or 3-ethylphenyl in compound **10** to 3-tolyl in compound **7**, 4-ethylphenyl in compound **11**, 4-ethoxyphenyl in compound **13**, 3,5-dimethoxyphenyl in compound **14**, 3,4,5- trimethoxyphenyl in compound **15** and 2-methyl-4-nitrophenyl in compound **16** diminshed the anticancer activity towards all the cell lines.
- The replacement of 3-ethylphenyl **10** by 4-ethylphenyl **11** lead to decrease the cytotoxic activity against A549, HepG2 and LoVo and MCF-7 cell lines.
- In addition the substitution of 3,5-dimethoxyphenyl **14** by 3,4,5-trimethoxyphenyl **15** reduces the anticancer activity towards all the cell lines.

### 2.4. Molecular docking analysis

Molecular docking simulations for promising compounds were also performed to obtain further understanding into differential cytotoxic action of synthesised compounds. The molecular docking procedure implemented in this study was validated as described by Al Ajmi and co-workers^32^. The cognate ligand, 1-(2-([(3S)-3-(aminomethyl)-3,4-dihydroisoquinolin-2(1H)-yl]carbonyl)phenyl)-4-chloro-5-methyl-N,N-diphenyl-1H-pyrazole-3-carboxamide (DRO), was extracted from the ligand**-**bound X-ray co-crystal structure of Bcl-2, and re-docked. The poses of the bound and re-docked ligand were compared, and the root mean square deviation (RMSD) was calculated. The RMSD of the re-docked DRO was found to be 0.3842 Å. Since the calculated RMSD was within the acceptable limit (2.0 Å), we were confident in adopting the docking protocol to predict the binding of compounds **6** and **10** with Bcl-2.

**Figure 8.**
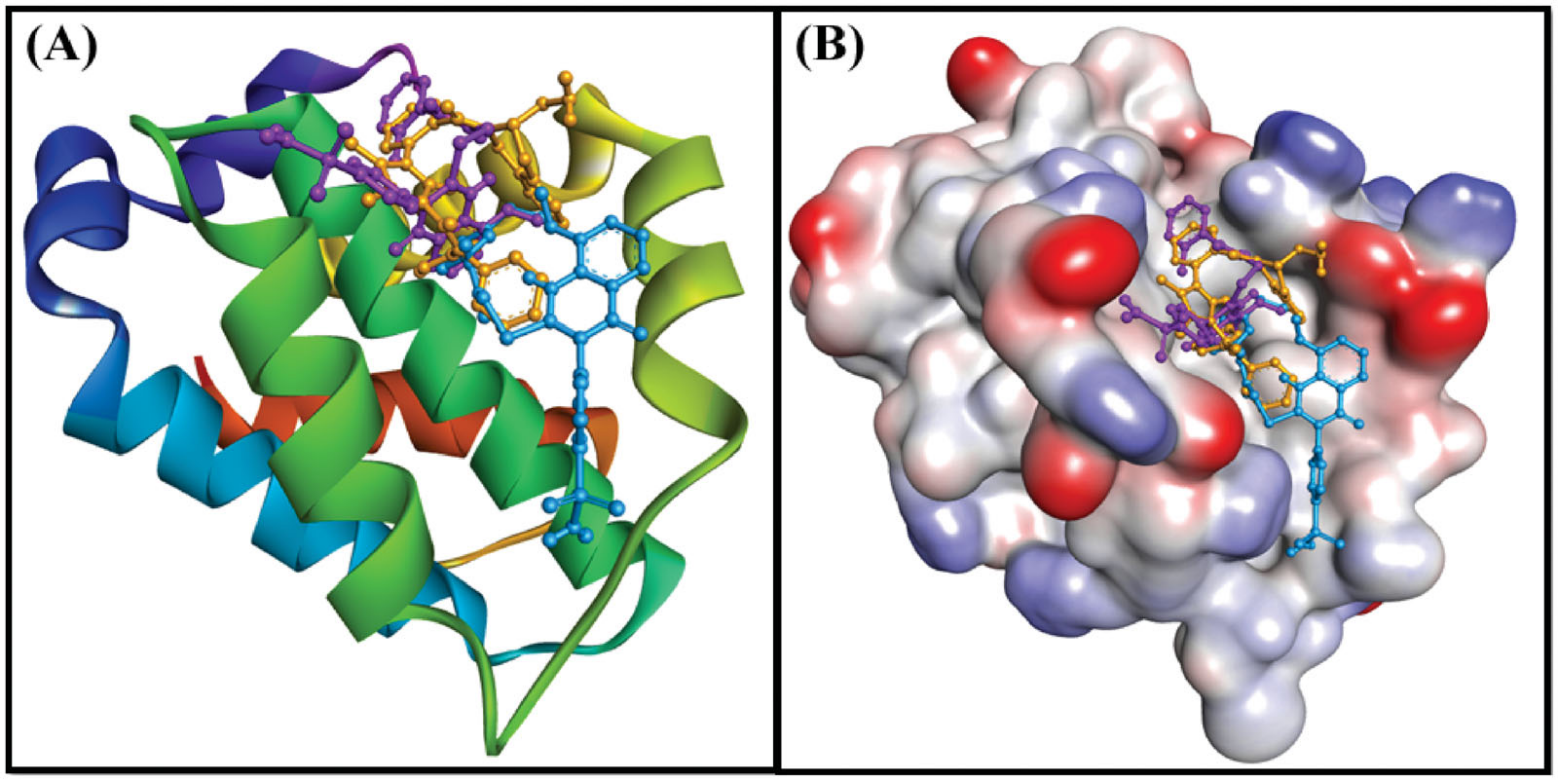
(A) 2D cartoon representation of the binding of DRO (control ligand), compound **6** and compound **10** at the binding site of the Bcl-2 protein, and (B) 3D representation of the binding of DRO (control ligand), compound **6** and compound **10** at the binding site of the Bcl-2 protein. DRO, compound **6** and compound **10** are represented by gold, purple and cyan sticks, respectively.

**Figure 9.**
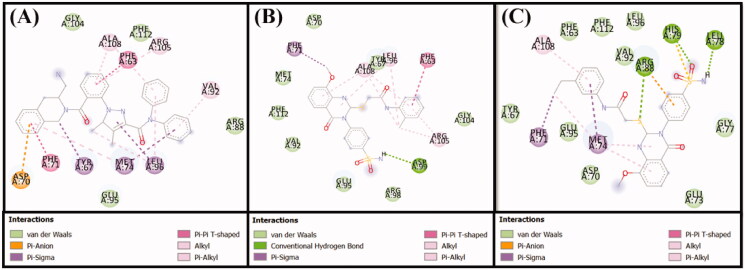
Molecular docking of (A) the control ligand DRO, (B) compound **6**, and (C) compound **10**, with the Bcl-2 protein.

Anti-apoptotic Bcl-2 protein overexpression is frequently linked to several types of cancer. Recently, a lot of attention on developing effective inhibitors to reduce the increased levels of this protein^33^^,^^34^. In this study, the molecular docking of compounds **6** and **10** with Bcl-2 was performed using Autodock 4.2, and the binding was compared with that of DRO (Table 2), (Figures 8 and 9). We found that both compounds **6** and **10** occupied a similar position at the Bcl-2 binding site as DRO (Figures 8 and 9). Analysis of the interactions between compound **6** and Bcl-2 revealed that the protein-ligand complex was stabilised by a hydrogen bond with Asp99, and nine hydrophobic interactions with Phe63, Phe71, Leu96, Arg105, and Ala108 (Table 2). The compound **6**-Bcl-2 complex was further stabilised by van der Waals’ interactions of the compound with several other residues, including Asp70, Met74, Val92, Glu95, Arg98, Gly104, and Phe112 (Figure 9(B)). Similarly, compound **10**-Bcl-2 complex was stabilised by three hydrogen bonds, with His79, Leu78, and Arg88, and an electrostatic interaction with Arg88. Compound **10** formed a further six hydrophobic interactions with Phe71, Met74, and Ala108 (Table 2). Compound **10** also formed a π-sulfur bond with His79 of Bcl-2. Additionally, the Phe63, Tyr67, Asp70, Glu73, Gly77, Val92, Glu95, Leu96, and Phe112 residues of Bcl-2 formed van der Waals’ interactions to stabilise the carvone-Bcl-2 complex (Figure 9(C)). In comparison, the DRO-Bcl-2 complex was stabilised by a hydrogen bond and an electrostatic interaction with the Asp70 residue (Figure 9(A)), as well as eleven hydrophobic interactions with Val92, Phe63, Tyr67, Phe71, Met74, Leu96, Arg105, and Ala108 (Table 2). The DRO-Bcl-2 complex was further stabilised by van der Waals’ interactions of DRO with the Arg88, Glu95, Gly104, and Phe112 residues of Bcl-2. The binding energies and the corresponding binding affinities of DRO, compounds **6** and **10** towards Bcl-2 were estimated to be −10.2 kcal mol**^−^**^1^ and 3.03 × 10^7^ M^−1^, −7.6 kcal mol**^−^**^1^ and 3.75 × 10^5^ M**^−^**^1^ and −7.9 kcal mol**^−^**^1^ and 6.23 × 10^5^ M**^−^**^1^, respectively (Table 2). It is interesting to note that the Phe63, Tyr67, Phe71, Met74, Leu96, and Ala108 residues of Bcl-2 showed interactions with both compound **6** and DRO, while the Phe63, Tyr67, Phe71, Met74, Val92, Leu96, and Ala108 residues showed interactions with compound **10** and DRO.

**Table 2.** Molecular docking of compounds **6** and **10** with Bcl-2

Donor atoms	Acceptor atom	Distance (Å)	Type of interaction	Estimated binding free energy, ΔG	Estimated binding affinity, *K*_d_
				(kcal mol^-1^)	(M^-1^)
*Bcl-2 and DRO* ^*^					
LIG: N	ASP70: OD2	2.9553	Conventional hydrogen bond	−10.2	3.03 × 10^7^
ASP70: OD2	LIG	4.8135	Electrostatic (π-anion)		
MET74: CE	LIG	3.5674	Hydrophobic (π-σ)		
LEU96: CD1	LIG	3.8681	Hydrophobic (π-σ)		
LIG: C	TYR67	3.5199	Hydrophobic (π-σ)		
PHE63	LIG	5.2880	Hydrophobic (π- π T-shaped)		
PHE71	LIG	4.9682	Hydrophobic (π- π T-shaped)		
LIG: C	LEU96	4.9539	Hydrophobic (alkyl)		
LIG	ARG105	5.2941	Hydrophobic (π-alkyl)		
LIG	ALA108	4.4414	Hydrophobic (π-alkyl)		
LIG	LEU96	5.1674	Hydrophobic (π-alkyl)		
LIG	ALA108	4.4935	Hydrophobic (π-alkyl)		
LIG	VAL92	5.0873	Hydrophobic (π-alkyl)		
*Bcl-2 and Compound 6*					
LIG:H	ASP99:OD2	2.8856	Conventional hydrogen bond	−7.6	3.75 × 10^5^
LIG:C	PHE71	3.7687	Hydrophobic (π-σ)		
A:PHE63	LIG	5.2080	Hydrophobic (π-π T-shaped)		
A:ALA108	LIG:C	3.5709	Hydrophobic (alkyl)		
LIG:C	LEU96	4.7454	Hydrophobic (alkyl)		
LIG:C	ARG105	4.6973	Hydrophobic (alkyl)		
LIG	ALA108	4.3570	Hydrophobic (π-alkyl)		
LIG	LEU96	5.3899	Hydrophobic (π-alkyl)		
LIG	ARG105	5.2307	Hydrophobic (π-alkyl)		
LIG	ALA108	4.9292	Hydrophobic (π-alkyl)		
*Bcl-2 and Compound 10*					
HIS79: HD1	LIG: O	2.0200	Conventional hydrogen bond	−7.9	6.23 × 10^5^
ARG88: HH12	LIG: S	2.9013	Conventional hydrogen bond		
LIG: H	LEU78: O	1.9625	Conventional hydrogen bond		
ARG88: NH1	LIG	3.3619	Electrostatic (π-cation)		
MET74: CE	LIG	3.8605	Hydrophobic (π-σ)		
LIG: C	PHE71	3.6647	Hydrophobic (π-σ)		
LIG: S	HIS79	5.3102	π-sulfur		
LIG: C	MET74	4.9968	Hydrophobic (alkyl)		
LIG	MET74	4.6108	Hydrophobic (π-alkyl)		
LIG	MET74	4.1329	Hydrophobic (π-alkyl)		
LIG	ALA108	4.8136	Hydrophobic (π-alkyl)		

*Chemically DRO is 1–(2-{[(3S)-3-(aminomethyl)-3,4-dihydroisoquinolin-2(1H)-yl] carbonyl} phenyl) -4-chloro-5-methyl-N, N-diphenyl-1H-pyrazole-3-carboxamide. It is the cognate ligand of Bcl-2.

## 3. Experimental

### 3.1. Chemistry

The melting points (MP; uncorrected) of the compounds were determined in an open capillary on a Gallen Kamp melting point apparatus (Sanyo Gallen Kamp, UK). Precoated silica gel plates *(Kieselgel* 0.25 mm, 60 F254, Merck, Germany) were used for thin-layer chromatography. A developing solvent system of chloroform/methanol (8:2) was used, and the spots were visualised using ultraviolet light. The IR spectra (KBr disc) were recorded using a Fourier transform-IR spectrophotometer (Perkin Elmer, USA). A nuclear magnetic resonance spectrophotometer (Bruker AXS Inc., Switzerland) was used for the ^1^H- and ^13 ^C-NMR experiments, operating at 500 MHz and 125.76 MHz, respectively. Chemical shifts are reported as δ-values (ppm) relative to tetramethylsilane (internal standard), using deuterated dimethyl sulfoxide (DMSO-d_6_) as the solvent. Elemental analyses were conducted using a model 2400 CHNSO analyser (Perkin Elmer, USA). All results were within ± 0.4% of the theoretical values. All reagents used were of analytical grade.

#### 4–(2-Mercapto-8-methoxy-4-oxoquinazolin-3(4H)-yl) benzenesulfonamide (4)

A mixture of 2-amino-3-methoxybenzoic acid **3** (1.67 g, 0.01 mol), 4- isothiocyanatobenzenesulphonamide **2** (2.14 g, 0.01 mol) in dioxan (30 ml) containing a catalytic amount of triethylamine was refluxed for 8 h and then left to cool. The solid product was collected by filtration and recrystallized from dioxan to yield **4**.

**4**: Yield: 92%. MP: 342–344 **°C**. IR (KBr, cm**^−^**^1^): 3321, 3262 (NH_2_), 3055 (aromatic), 1691 (CO), 1620 (CN), 1381, 1156 (SO_2_). ^1^H-NMR (DMSO-d_6_,ppm): 3.9 (*s*, 3H, OCH_3_), 7.0–7.7 (*m*, 5H, Ar-H + SO_2_NH_2_), 7.8, 8.0 (2d, 4H, *J* = 7.1, 7.0 Hz, AB system), 12.3 (*s*, 1H, SH). ^13 ^C-NMR (DMSO-d_6_,ppm): 55.7, 116.2, 118.6, 119.8, 120. 6 (2), 127.0, 128.9 (2), 130.1, 134.6, 135.3, 150.4, 160.5, 160.9. MS (m/z, %): 363 (M^+^) (14.54), 155 (100). Anal. Calcd. for C_15_H_13_N_3_O_4_S_2_ (363.41): C, 49.57; H, 3.61; N, 11.56. Found: C, 49.22; H, 3.86; N, 11.31.

#### 3.1.1. General procedure for the synthesis of 3, 4-dihydroquinazolin-sulphonamide derivatives (5–18)

Anhydrous K_2_CO_3_ (1.38 g) was added to a mixture of **4** (3.63 g, 0.01 mol) and 2-chloro-*N*-substituted acetamide derivatives (0.012 mol) in dry acetone (50 ml) and stirred at room temperature for 8 h. The resulting solid was then collected by filtration and crystallised from ethanol to yield compounds **5–18**.

#### 2-((8-Methoxy-4-oxo-3–(4-sulphamoylphenyl)-3,4-dihydroquinazolin-2-yl)thio)-N-phenyl-acetamide (5)

**5**: Yield: 77%. MP: > 350 °C. IR (KBr, cm**^−^**^1^): 3324, 3291, 3164 (NH_2_, NH), 3056 (aromatic), 2967, 2845 (aliphatic), 1687, 1663 (2 CO), 1619 (CN), 1385, 1155 (SO_2_). ^1^H-NMR (DMSO-d_6_,ppm): 3.8 (*s*, 3H, OCH_3_), 4.0 (*s*, 2H, CH_2_), 7.3, 7.5 (2d, 4H, *J* = 7.3, 7.2 Hz, AB system), 7.6 − 8.0 (*m*, 10H, Ar-H + SO_2_NH_2_), 10.3 (*s*, 1H, NH). ^13 ^C-NMR (DMSO-d_6_,ppm): 31.3, 56.6, 116.5, 117.4, 118.6 (2), 119.6, 124.8 (2), 126.9, 127.4, 129.2 (2), 130.3 (2), 130.7 (2), 143.1, 143.9, 151.3, 157.8, 159.2, 175.6. MS (*m*/*z*, %): 496 (M^+^) (32.21), 77 (100). Anal. Calcd. for C_23_H_20_N_4_O_5_S_2_ (496.56): C, 55.63; H, 4.06; N, 11.28. Found: C, 55.87; H, 4.19; N, 11.49.

#### 2-((8-Methoxy-4-oxo-3-(4-sulphamoylphenyl)-3,4-dihydroquinazolin-2-yl)thio)-N-(o-tolyl) acetamide (6)

**6**: Yield: 86%. MP: > 350 °C. IR (KBr, cm**^−^**^1^): 3421, 3365, 3154 (NH_2_, NH), 3077 (aromatic), 2958, 2837 (aliphatic), 1687, 1660 (2 CO), 1618 (CN), 1388, 1156 (SO_2_). ^1^H-NMR (DMSO-d_6_,ppm): 2.4 (*s*, 3H, CH_3_), 3.8 (*s*, 3H, OCH_3_), 3.9 (*s*, 2H, CH_2_), 7.3, 7.4 (2d, 4H, *J* = 6.8, 6.7 Hz, AB system), 7.5 − 7.9 (*m*, 9H, Ar-H + SO_2_NH_2_), 11.9 (*s*, 1H, NH). ^13 ^C-NMR (DMSO-d_6_,ppm): 15.0, 29.3, 56.9, 116.7, 117.3, 118.6, 125.2 (2), 126.9 (2), 130.2 (2), 130.3 (2), 131.2 (2), 133.1 (2), 142.8 (2), 144.4, 160.0, 160.2, 175.8. MS (*m*/*z*, %): 510 (M^+^) (33.32), 91 (100). Anal. Calcd. for C_24_H_22_N_4_O_5_S_2_ (510.59): C, 56.46; H, 4.34; N, 10.97. Found: C, 56.18; H, 4.05; N, 11.17.

#### 2-((8-Methoxy-4-oxo-3-(4-sulphamoylphenyl)-3,4-dihydroquinazolin-2-yl)thio)-N-(m-tolyl) acetamide (7)

**7**: Yield: 78%. MP: > 350 °C. IR (KBr, cm**^−^**^1^): 3311, 3234, 3176 (NH_2_, NH), 3065 (aromatic), 2912, 2845 (aliphatic), 1691, 1665 (2 CO), 1621 (CN), 1383, 1155 (SO_2_). ^1^HNMR (DMSO-d_6_,ppm): 2.2 (s, 3H, CH_3_), 3.8 (s, 3H, OCH_3_), 3.9 (*s*, 2H, CH_2_), 7.3, 7.5 (2d, *J* = 8.1, 8.2 Hz, 4H, AB system), 7.6 − 7.9 (*m*, 9H, Ar-H + SO_2_NH_2_), 10.0 (*s*, 1H, NH). ^13 ^C-NMR (DMSO-d_6_,ppm): 21.2, 29.3, 56.9, 116.6, 117.4, 118.6 (2), 124.9, 126.9 (2), 130.3 (2), 130.8 (3), 131.4 (2), 135.6, 136.0, 143.0, 151.3, 158.6, 160.7, 175.7. MS (*m*/*z*, %): 510 (M^+^) (11.62), 77 (100). Anal. Calcd. for C_24_H_22_N_4_O_5_S_2_ (510.59): C, 56.46; H, 4.34; N, 10.97. Found: C, 56.66; H, 4.65; N, 10.64.

#### 2-((8-Methoxy-4-oxo-3-(4-sulphamoylphenyl)-3,4-dihydroquinazolin-2-yl)thio)-N-(p-tolyl) acetamide (8)

**8**: Yield: 90%; MP: > 350 °C. IR (KBr, cm**^−^**^1^): 3345, 3256, 3180 (NH_2_, NH), 3097 (aromatic), 2967, 2823 (aliphatic), 1690, 1667 (2 CO), 1622 (CN), 1381, 1165 (SO_2_). ^1^H-NMR (DMSO-d_6_,ppm): 2.3 (*s*, 3H, CH_3_), 3.8 (*s*, 3H, OCH_3_), 3.9 (*s*, 2H, CH_2_), 7.3, 7.4 (2d, 4H, *J* = 8.0, 7.9 Hz, AB system-tolyl), 7.5, 7.6 (2d, *J* = 7.4, 7.3 Hz, 4H, AB system), 7.7 − 7.9 (*m*, 9H, Ar-H + SO_2_NH_2_), 10.4 (*s*, 1H, NH). ^13 ^C-NMR (DMSO-d_6_,ppm): 20.6, 31.2, 56.9, 116.6, 117.4, 118.6 (2), 119.6, 125.0 (2), 126.9, 129.5 (2), 130.3 (2), 130.4, 136.7, 137.8, 137.9, 143.0, 150.7, 156.3, 160.1, 175.7. MS (*m*/*z*, %): 510 (M^+^) (12.98), 154 (100). Anal. Calcd. for C_24_H_22_N_4_O_5_S_2_ (510.59): C, 56.46; H, 4.34; N, 10.97. Found: C, 56.22; H, 4.11; N, 10.67.

#### N-(2-Ethylphenyl)-2-((8-methoxy-4-oxo-3-(4-sulphamoylphenyl)-3,4-dihydroquinazolin-2-yl)thio) acetamide (9)

**9**: Yield: 92%. MP: 288 − 290 °C. IR (KBr, cm**^−^**^1^): 3412, 3387, 3178 (NH_2_, NH), 3100 (aromatic), 2988, 2827 (aliphatic), 1685, 1666 (2 CO), 1612 (CN), 1391, 1155 (SO_2_). ^1^H-NMR (DMSO-d_6_,ppm): 1.0 (*t*, 3H, *J* = 6.6 Hz, CH_3_ ethyl), 2.5 (*q*, 2H, *J* = 8.3 Hz, CH_2_ ethyl), 3.8 (*s*, 3H, OCH_3_), 4.1 (*s*, 2H, CH_2_), 7.7, 7.8 (2d, 4H, *J* = 6.9, 6.8 Hz, AB system), 7.1 − 7.6 (*m*, 9H, Ar-H + SO_2_NH_2_), 9.6 (*s*, 1H, NH). ^13 ^C-NMR (DMSO-d_6_,ppm): 14.5, 24.0, 31.0, 56.7, 116.3, 118.1, 120.9, 126.4 (2), 126.5, 127.1, 127.5, 128.9 (2), 130.7 (2), 135.2 (2), 138.0, 138.5, 139.0, 153.7, 155.5, 161.0, 166.5. MS (*m*/*z*, %): 524 (M^+^) (54.93), 121 (100). Anal. Calcd. for C_25_H_24_N_4_O_5_S_2_ (524.61): C, 57.24; H, 4.61; N, 10.68. Found: C, 57.51; H, 4.86; N, 10.46.

#### N-(3-Ethylphenyl)-2-((8-methoxy-4-oxo-3-(4-sulphamoylphenyl)-3,4-dihydroquinazolin-2-yl)thio) acetamide (10)

**10**: Yield: 86%. MP: 292 − 294 °C. IR (KBr, cm**^−^**^1^): 3345, 3300, 3152 (NH_2_, NH), 3078 (aromatic), 2945, 2856 (aliphatic), 1689, 1661 (2 CO), 1618 (CN), 1390, 1165 (SO_2_). ^1^H-NMR (DMSO-d_6_,ppm): 1.1 (*t*, 3H, *J* = 6.5 Hz, CH_3_ ethyl), 2.5 (*q*, 2H, *J* = 6.8 Hz, CH_2_ ethyl), 3.9 (*s*, 3H, OCH_3_), 4.0 (*s*, 2H, CH_2_), 7.9, 8.0 (2d, 4H, *J* = 7.0, 7.1 Hz, AB system), 7.2 − 7.7 (*m*, 9H, Ar-H + SO_2_NH_2_), 10.2 (*s*, 1H, NH). ^13 ^C-NMR (DMSO-d_6_,ppm): 15.9, 28.7 (2), 56.7, 117.0, 118.1, 118.9, 120.9, 123.4, 126.9 (2), 127.1, 127.4, 129.1, 130.3 (2), 130.7, 138.0, 139.1, 139.3, 144.7, 153.7, 155.6, 161.0, 166.0. MS (*m*/*z*, %): 524 (M^+^) (22.76), 231 (100). Anal. Calcd. for C_25_H_24_N_4_O_5_S_2_ (524.61): C, 57.24; H, 4.61; N, 10.68. Found: C, 57.02; H, 4.35; N, 10.89.

#### N-(4-Ethylphenyl)-2-((8-methoxy-4-oxo-3-(4-sulphamoylphenyl)-3,4-dihydroquinazolin-2-yl)thio) acetamide (11)

**11**: Yield: 88%. MP: 280 − 282 °C. IR (KBr, cm**^−^**^1^): 3400, 3363, 3145 (NH_2_, NH), 3075 (aromatic), 2945, 2867 (aliphatic), 1687, 1665 (2 CO), 1619 (CN), 1378, 1163 (SO_2_). ^1^H-NMR (DMSO-d_6_,ppm): 1.1 (*t*, 3H, *J* = 8.4 Hz, CH_3_ ethyl), 2.5 (*q*, 2H, *J* = 7.9 Hz, CH_2_ ethyl), 3.8 (*s*, 3H, OCH_3_), 4.0 (*s*, 2H, CH_2_), 7.1, 7.4 (2d, 4H, *J* = 7.6, 7.7 Hz, AB system), 7.7 − 8.0 (2d, 4H, *J* = 7.4, 7.5 Hz, AB system), 7.3 − 7.7 (*m*, 5H, Ar-H + SO_2_NH_2_), 10.3 (*s*, 1H, NH). ^13 ^C-NMR (DMSO-d_6_,ppm): 16.1, 28.0 (2), 56.7, 116.4, 118.1, 119.7 (2), 120.9, 127.4 (2), 128.4 (2), 130.7 (3), 137.0, 138.0, 139.3, 139.4, 146.2, 153.7, 155.6, 161.0, 165.8. MS (*m*/*z*, %): 524 (M^+^) (8.05), 106 (100). Anal. Calcd. for C_25_H_24_N_4_O_5_S_2_ (524.61): C, 57.24; H, 4.61; N, 10.68. Found: C, 57.49; H, 4.78; N, 10.87.

#### 2-((8-Methoxy-4-oxo-3-(4-sulphamoylphenyl)-3,4-dihydroquinazolin-2-yl)thio)-N-(p-methoxyphenyl) acetamide (12)

**12**: Yield: 66%. MP: 296 − 298 °C. IR (KBr, cm**^−^**^1^): 3445, 3343, 3129 (NH_2_, NH), 3078 (aromatic), 2976, 2845 (aliphatic), 1677, 1657 (2 CO), 1621 (CN), 1376, 1176 (SO_2_). ^1^H-NMR (DMSO-d_6_,ppm): 3.8, 3.9 (2 s, 6H, 2 OCH_3_), 4.0 (*s*, 2H, CH_2_), 6.8, 7.4 (2d, 4H, *J* = 6.7, 6.6 Hz, AB system), 7.6, 8.0 (2d, 4H, *J* = 7.2, 7.3 Hz, AB system), 7.5–7.9 (*m*, 5H, Ar-H + SO_2_NH_2_), 10.2 (s, 1H, NH). ^13 ^C-NMR (DMSO-d_6_,ppm): 32.3, 55.6, 56.9, 114.3 (2), 116.4, 118.1, 120.9, 121.2 (2), 126.9 (2), 127.4, 130.3 (2), 130.7, 132.4, 138.0, 139.1, 153.7, 155.6, 155.8, 161.0, 165.5. MS (*m*/*z*, %): 526 (M^+^) (5.43), 165 (100). Anal. Calcd. for C_24_H_22_N_4_O_6_S_2_ (526.58): C, 54.74; H, 4.21; N, 10.64. Found: C, 54.49; H, 4.00; N, 10.40.

#### N-(p-Ethoxyphenyl)-2-((8-methoxy-4-oxo-3-(4-sulphamoylphenyl)-3,4-dihydroquin-azolin-2-yl)thio) acetamide (13)

**13**: Yield: 69%. MP: 300 − 302 °C. IR (KBr, cm**^−^**^1^): 3410, 3376, 3161 (NH_2_, NH), 3058 (aromatic), 2978, 2845 (aliphatic), 1687, 1661 (2 CO), 1613 (CN), 1386, 1155 (SO_2_). ^1^H-NMR (DMSO-d_6_,ppm): 1.3 (*t*, 3H, *J* = 8.5 Hz, CH_3_), 3.8 (*s*, 3H, OCH_3_), 4.0 (*s*, 2H, SCH_2_), 4.1 (*q*, 2H, *J* = 8.0 Hz, CH_2_), 6.8, 7.3 (2d, 4H, *J* = 7.7, 7.8 Hz, AB system), 7.5, 7.7 (2d, 4H, *J* = 7.0, 7.2 Hz, AB system), 7.0–7.8 (*m*, 5H, Ar-H + SO_2_NH_2_), 10.3 (*s*, 1H, NH). ^13 ^C-NMR (DMSO-d_6_,ppm): 18.2, 28.9, 55.8, 56.9, 115.6 (2), 115.7, 118.9, 119.9, 120.9 (2), 126.3 (2), 126.8, 130.3 (2), 130.6, 133.7, 133.9, 138.3, 154.6, 155.8, 157.7, 160.0, 175.4. MS (*m*/*z*, %): 540.61 (M^+^) (22.09), 264 (100). Anal. Calcd. for C_25_H_24_N_4_O_6_S_2_ (540.61): C, 55.54; H, 4.47; N, 10.36. Found: C, 55.27; H, 4.68; N, 10.09.

#### N-(3,5-Dimethoxyphenyl)-2-((8-methoxy-4-oxo-3-(4-sulphamoylphenyl)-3,4-dihydroq-uinazolin-2-yl)thio) acetamide (14)

**14**: Yield: 76%. MP: 240 − 242 °C. IR (KBr, cm**^−^**^1^): 3460, 3311, 3178 (NH_2_, NH), 3071 (aromatic), 2971, 2855 (aliphatic), 1691, 1660 (2 CO), 1620 (CN), 1377, 1161 (SO_2_). ^1^H-NMR (DMSO-d_6_,ppm): 3.6, 3.8 (2 s, 9H, 3 OCH_3_), 4.0 (*s*, 2H, SCH_2_), 6.2 (*s*, 1H, CH), 6.80, 6.82 (2 s, 2H, Ar-H), 7.4, 7.5 (2d, 4H, *J* = 6.2, 6.1 Hz, AB system), 7.5–7.9 (*m*, 6H, Ar-H + SO_2_NH_2_ + NH). ^13 ^C-NMR (DMSO-d_6_,ppm): 31.2, 55.5, 56.8 (2), 95.9, 97.9 (2), 116.4, 118.1, 120.9, 127.0 (2), 127.1, 129.3 (2), 131.0, 131.3, 138.0, 141.0, 153.7, 160.9, 161.0 (2), 166.2 (2). MS (*m*/*z*, %): 556 (M^+^) (22.09), 264 (100). Anal. Calcd. for C_25_H_24_N_4_O_7_S_2_ (556.61): C, 53.95; H, 4.35; N, 10.07. Found: C, 54.27; H, 4.69; N, 10.29.

#### 2-((8-Methoxy-4-oxo-3-(4-sulphamoylphenyl)-3,4-dihydroquinazolin-2-yl)thio)-N-(3,4,5-trimethoxyphenyl) acetamide (15)

**15**: Yield: 86%. MP: 290 − 292 °C. IR (KBr, cm**^−^**^1^): 3356, 3256, 3176 (NH_2_, NH), 3100 (aromatic), 2942, 2856 (aliphatic), 1690, 1667 (2 CO), 1623 (CN), 1367, 1152 (SO_2_). ^1^H-NMR (DMSO-d_6_,ppm): 3.71, 3.90, 3.92 (3 s, 12H, 4 OCH_3_), 4.08 (*s*, 2H, SCH_2_), 6.95 (*s*, 2H, Ar-H), 7.45, 7.48 (2d, 4H, *J* = 7.4, 7.2 Hz, AB system), 7.5 − 8.0 (*m*, 5H, Ar-H + SO_2_NH_2_), 10.2 (*s*, 1H, NH). ^13 ^C-NMR (DMSO-d_6_,ppm): 32.1, 56.1, 56.8, 56.9, 60.5, 97.3 (2), 116.5, 118.1, 120.9, 126.7 (2), 127.0, 127.4 (2), 130.3, 133.9, 135.5, 138.0, 139.0, 153.1, 153.7 (2), 155.4, 161.0, 165.9. MS (*m*/*z*, %): 586.64 (M^+^) (33.32), 91 (100). Anal. Calcd. for C_26_H_26_N_4_O_8_S_2_ (586.64): C, 53.23; H, 4.47; N, 9.55. Found: C, 52.98; H, 4.15; N, 9.21.

#### 2-((8-Methoxy-4-oxo-3-(4-sulphamoylphenyl)-3,4-dihydroquinazolin-2-yl)thio)-N-(2-methyl-4-nitrophenyl) acetamide (16)

**16**: Yield: 86%. MP: > 350 °C. IR (KBr, cm**^−^**^1^): 3421, 3365, 3154 (NH_2_, NH), 3077 (aromatic), 2958, 2837 (aliphatic), 1687, 1660 (2 CO), 1618 (CN), 1388, 1156 (SO_2_). ^1^H-NMR (DMSO-d_6_,ppm): 2.3 (*s*, 3H, CH_3_), 3.9 (*s*, 3H, OCH_3_), 4.1 (*s*, 2H, CH_2_), 6.8, 7.4 (2d, 4H, *J* = 7.6, 7.7 Hz, AB system), 7.51–7.9 (*m*, 8H, Ar-H + SO_2_NH_2_), 10.1 (*s*, 1H, NH). ^13 ^C-NMR (DMSO-d_6_,ppm): 19.3, 30.3, 58.6, 109.4, 116.7, 117.3, 120.6, 121.6, 122.7 (2), 124.7, 127.6, 128.7 (2), 129.1, 133.6, 134.6, 135.7, 141.9, 142.3, 150.7, 160.8, 161.2, 165.6. MS (*m*/*z*, %): 555 (M^+^) (1.54), 163 (100). Anal. Calcd. for C_24_H_21_N_5_O_7_S_2_ (555.58): C, 51.88; H, 3.81; N, 12.61. Found: C, 51.56; H, 4.09; N, 12.32.

#### 2-((8-Methoxy-4-oxo-3-(4-sulphamoylphenyl)-3,4-dihydroquinazolin-2-yl)thio)-N-(2-methyl-6-nitrophenyl) acetamide (17)

**17**: Yield: 71%. MP: > 350 °C. IR (KBr, cm**^−^**^1^): 3400, 3318, 3162 (NH_2_, NH), 3065 (aromatic), 2994, 2843 (aliphatic), 1684, 1666 (2 CO), 1622 (CN), 1378, 1155 (SO_2_). ^1^H-NMR (DMSO-d_6_,ppm): 2.2 (*s*, 3H, CH_3_), 3.8 (*s*, 3H, OCH_3_), 4.1 (*s*, 2H, CH_2_), 7.4, 7.6 (2d, 4H, *J* = 7.0, 6.9 Hz, AB system), 7.5 − 7.9 (*m*, 8H, Ar-H + SO_2_NH_2_), 10.2 (*s*, 1H, NH). ^13 ^C-NMR (DMSO-d_6_,ppm): 18.0, 27.9, 56.7, 116.4, 118.1, 120.0, 122.6 (2), 127.1, 127.4, 127.5, 128.7, 130.7 (2), 135.4, 137.7, 137.9, 130.0 (2), 145.9, 153.7, 155.2, 161.0, 166.4. MS (*m*/*z*, %): 555 (M^+^) (8.54), 212 (100). Anal. Calcd. for C_24_H_21_N_5_O_7_S_2_ (555.58): C, 51.88; H, 3.81; N, 12.61. Found: C, 66.56; H, 3.61; N, 12.85.

#### N-(2,4-Dinitrophenyl)-2-((8-methoxy-4-oxo-3-(4-sulphamoylphenyl)-3,4-dihydro-quinazo-lin-2-yl)thio) acetamide (18)

**18**: Yield: 86%. MP: > 350 °C. IR (KBr, cm**^−^**^1^): 3365, 3312, 3177 (NH_2_, NH), 3082 (aromatic), 2991, 2863 (aliphatic), 1689, 1664 (2 CO), 1624 (CN), 1378, 1158 (SO_2_). ^1^H-NMR (DMSO-d_6_,ppm): 3.92 (*s*, 3H, OCH_3_), 3.95 (*s*, 2H, CH_2_), 7.41, 7.63 (2d, 4H, *J* = 8.4, 8.5 Hz, AB system), 7.56 − 7.93 (*m*, 8H, Ar-H + SO_2_NH_2_), 8.67 (*s*, 1H, NH). ^13 ^C-NMR (DMSO-d_6_,ppm): 29.6, 56.9, 116.6, 117.4, 118.6, 125.0, 126.9 (2), 130.3 (2), 130.7 (2), 136.1 (2), 136.3 (2), 137.6, 143.0, 144.0, 151.3, 160.2, 162.4, 175.7. MS (*m*/*z*, %): 586 (M^+^) (17.62), 187 (100). Anal. Calcd. for C_23_H_18_N_6_O_9_S_2_ (586.55): C, 47.10; H, 3.09; N, 14.33. Found: C, 47.36; H, 3.31; N, 14.06.

### 3.2. Biological evaluation

#### 3.2.1. Cytotoxicity assay

The MTT assay was performed to test the antiproliferative activity of the synthesised compounds, as per the methodology described by Alqahtani and co-workers^35^. Briefly, A549, HepG2, LoVo, MCF-7 and HUVEC cell lines were seeded in 96**-**cell culture plates (5 × 10^4^ per well) and allowed 24 h for adherence. The cells were then treated with different concentrations of each compound, and 5-fluorouracil was used as a positive control. Following the treatment period (48 h), 10 µL of MTT solution (5 mg/mL) was added to each well and the plates were incubated at 37 °C for 2–4 h. Isopropanol (100 µL) acidified with 0.1 N HCl was added to solubilise the formazan products and the plate was kept on a shaker for 10 min. The optical density of each mixture was measured at 570 nm using an enzyme-linked immunosorbent assay plate reader (ELISA plate reader, Bio-Tek, USA). The concentrations of tested compounds required to inhibit cell growth by 50% (IC_50_) were calculated using a dose-response curve. Cell survival was calculated using the following equation:

Cell survival (%)=(OD of treated sample)/(OD of untreated sample)×100

#### 3.2.2. Cell cycle analysis

Cell cycle analysis was conducted as previously described by Alqahtani and co-workers^35^. Briefly, MCF-7 cells were seeded in 6-well plates and incubated for 24 h before the addition of various concentrations (10, 20 and 30 µM) of compounds **6** and **10**. After incubating for 24 h, the cells were harvested, washed, and resuspended in PBS. The cells were fixed with 70% ethanol at 4 °C for 4 h. The cells were then incubated with RNase (100 µg/mL) and PI (50 µg/mL) for 30 min in the dark. Flow cytometry analysis was performed using Cytomics FC 500 (Beckman Coulter, Brea, CA, USA).

#### 3.2.3. Quantification of apoptosis by flow cytometric analysis

Apoptosis was measured using the Annexin V/PI detection kit (BioLegend, CA, USA), as per the manufacturer’s instructions. Briefly, MCF-7 cells were treated with different concentrations (10, 20 and 30 µM) of compounds **6** and **10** for 24 h. After treatment, the detached and adherent cells were collected, washed, and resuspended in 100 µL of 1X binding buffer. Then, 5 µL of Annexin V and 5 µL of PI were added to the resuspended cells. After incubating for 15 min in the dark, 400 µL of binding buffer was added to each tube and cell samples were analysed using Cytomics FC 500 (Beckman Coulter, Brea, CA, USA). Data collection and analysis were conducted using the CXP software V. 3.0 (Aspect, Phoenix, AZ, USA).

#### 3.2.4. Rt-pcr

MCF-7 cells were cultured with different concentrations (10, 20 and 30 µM) of compounds **6** and **10** for 24 h, and the untreated cells were used as a control. The cells were lysed using Trizol reagent (Invitrogen, USA). Total RNA was extracted as per the manufacturer’s instructions. Total RNA was quantitated by taking 2 µL of resuspended RNA on a Nano drop spectrophotometer (Thermo Scientific, USA) and reverse transcribing an equal amount (1 µg) of RNA to make complementary deoxyribonucleic acid (cDNA) using a Super Script VILO cDNA synthesis Kit (Invitrogen), as per the manufacturer’s protocol, in a final volume of 20 µL. The mixture was incubated at 42 °C for 1 h. The generated cDNA (2 µL) was used to assess the mRNA expression of apoptotic genes, including caspase-7, Bax, Bcl-2, and p53. The actin gene was used as an internal control. RT-PCR was performed using 5x Firepol Master Mix ready to load (Solis BioDyne, Tartu, Estonia), as per the manufacturer’s instructions. The specific primer sets used in this study are listed in Table 3. The program was run as follows: initial denaturation at 95 °C for 5 min, denaturation at 95 °C for 30 s, annealing at 55 °C for 45 s, elongation at 72 °C for 45 s (30 cycles), and final extension at 72 °C for 10 min. The RT-PCR products were electrophoresed on a 1.2% agarose gel containing ethidium bromide, and the gel was imaged on a Licor machine.

**Table 3.** List of primer sequences used for reverse transcriptase-polymerase chain reaction in this study. F: Forward, R: Reverse

Genes	Primer Sequence
**β-actin**	**F: 5′ –** CATCGTGATGGACTCTGGTG **− 3′**
	**R: 5′ –** TTTGATGTCACGCACGATTT **− 3′**
**Caspase-7**	**F: 5′ –** AGTGACAGGTATGGGCGTTC **− 3′**
	**R: 5′ –** TCCATGGCTTAAGAGGATGC **− 3′**
**Bcl2**	**F: 5′ –** TGATGCCTTCTGTGAAGCAC **− 3′**
	**R: 5′ –** ACAGGCGGAGCTTCTTGTAA **− 3′**
**Bax**	**F: 5′ –**TTTGCTTCAGGGTTTCATCC **− 3′**
	**R: 5′ –** ATCCTCTGCAGCTCCATGTT **− 3′**
**P53**	**F: 5′ –**TGGCTCTGACTGTACCACCATCC**– 3′**
	**R: 5′-**CAGCTCTCGGAACATCTCGAAGC**– 3′**

#### 3.2.5. Western blot analysis

MCF-7 cells were treated with compounds **6** and **10** for 24 h. The cells were then harvested and washed twice with 1x PBS, by adding lysis buffer containing 20 mM Tris (pH 7.5), 150 mM NaCl, 1 mM sodium ethylenediaminetetraacetic acid, 1 mM ethylene glycol-bis (β-aminoethyl ether)-N,N,N′,N′-tetraacetic acid, 1% Triton X100, 1 µg/mL leupeptin, and 100 µM phenylmethylsulphonyl fluoride, to prepare the total cell extract. The lysate was cooled on ice for 1 h and clarified by centrifugation at 13000 rpm at 4 °C for 15 min. The supernatants were collected. The protein concentrations of all the samples were determined using Bradford reagent (BioRad, Hercules, CA, USA). For western blot analysis, 30 − 35 µg of the total protein was loaded onto 10% sodium dodecyl sulphate-polyacrylamide gel and then transferred to a polyvinylidene fluoride membrane. The membrane was blocked with 5% bovine serum albumin in 0.1% Tween-tris-buffered saline (TBST) buffer for 2 h at room temperature, and then washed thrice with TBST. The membrane was incubated with primary antibodies for Bcl-2, p53, caspase7, Bax, and β-actin (1:200, Santa Cruz Biotechnology). To ensure equal loading, an anti-actin antibody was also used. The membrane was incubated with horseradish peroxidase-conjugated anti-mouse secondary antibody diluted 1: 1000 for 1 h at room temperature. After incubation, the membrane was washed thrice with TBST. It was then ready for immunodetection; the membrane was incubated with enhanced chemiluminescence western blotting detection reagents (Amersham, Pharmacia Biotech Inc., Piscataway, NJ, USA) and bands were obtained on exposing to X-ray films (Amersham).

### 3.3. Molecular docking

The potential of the two most promising compounds **6** and **10** to inhibit Bcl-2 was evaluated in molecular docking experiments, conducted as described by Al-Shabib and co-workers^32^. The 3 D coordinates of Bcl-2 were retrieved from the PDB-RCSB databank (PDB ID: **2W3L**). The X-ray crystal structure of DRO-bound Bcl-2 has previously been solved at a resolution of 2.10 Å^36^. Prior to molecular docking, the protein was pre-processed to remove crystallographic water molecules and any other heteroatoms, add hydrogen atoms, assign proper bond order, and define rotatable bonds, as previously described by Rehman and co-workers^37^. A network of H-bonds was created, and the energy of the protein was minimised using the Merck Molecular Force Field. A grid box of 27 × 30 × 25 Å, centred at 39 × 28 × −12 Å, with 0.375 Å spacing was defined as a conformation search space for the binding of ligands to Bcl-2. Finally, molecular docking between ligands and proteins was performed using Autodock 4.2 (Scripps Research, San Diego, CA, USA), as previously described by Rabbani and co-workers^38^. Molecular docking was performed using the Lamarckian Genetic Algorithm and Solis and Wets local search methods. The initial torsions, positions, and orientations of the ligands were set randomly. For each docking run, a maximum of 2.5 × 10^6^ calculations was enumerated after setting a population size of 150 and a translational step of 0.2 Å. The quaternion and torsion steps were set to 5. Discovery Studio (BIOVIA, San Diego, CA, USA) was used to analyse the docking results and prepare the figures. Binding affinities of compounds **6** and **10** for Bcl-2 were determined from their respective binding energies (Δ*G*), using the following relationship^39^.

$$\Delta G=\;-\mathrm{RTln}K_{d}$$

Here, *R* is the Boltzmann gas constant (1.987 cal mol**^−^**^1^ K**^−^**^1^) and *T* is the temperature (298 K).

### 3.4. Statistical analysis

Statistical analysis was performed using OriginPro 8.5 software (OriginLab, Northampton, MA, USA). Data are shown as the mean ± standard deviation. Differences were analysed using Student’s t-test, and were considered statistically significant if *p* < 0.05. *p < 0.05, **p < 0.01, ***p < 0.001.

## 4. Conclusion

In conclusion, a new series of quinazoline-sulphonamide derivatives **5–18** were synthesised and evaluated *in vitro* for their antiproliferative activity. Most of the prepared compounds were found to exhibit remarkable cytotoxicity in the MCF-7 breast cancer cell line. Compounds **6** and **10** were found to be the most promising. Flow cytometry data revealed that compounds **6** and **10** arrested the cell cycle of MCF-7 cells in the sub-G1 and induced apoptosis in cell death mode. Furthermore, changes in the expression of apoptosis-related markers at the gene and protein level were also indicative of apoptotic activity. The 2-tolyl derivative **6** and the 3-ethylphenyl derivative **10** downregulated the expression of B-cell lymphoma-2 (Bcl-2), while increasing that of p53, Bcl-2-like protein 4, and caspase-7, at the mRNA and protein levels. Molecular docking of compounds **6** and **10** also suggested that they possess good binding affinity for Bcl-2. Overall, the findings suggest that compounds **6** and **10** both possess promising anti-proliferative activity. These molecules may be further modified to develop more selective, clinically useful analogues.
